# Cold storage reveals distinct metabolic perturbations in processing and non-processing cultivars of potato (*Solanum tuberosum* L.)

**DOI:** 10.1038/s41598-020-63329-5

**Published:** 2020-04-14

**Authors:** Sagar S. Datir, Saleem Yousf, Shilpy Sharma, Mohit Kochle, Ameeta Ravikumar, Jeetender Chugh

**Affiliations:** 1 Department of Biotechnology, Savitribai Phule Pune University, Ganeshkhind, Pune, 411007 India; 2 Present Address: Biology Department, Biosciences Complex, Queen’s University, Kingston, Ontario K7L 3N6 Canada; 3 Department of Chemistry, Indian Institute of Science Education and Research, Pune, 411008 India; 4 Institute of Bioinformatics and Biotechnology, Savitribai Phule Pune University, Ganeshkhind, Pune, 411007 India; 5 Department of Biology, Indian Institute of Science Education and Research, Pune, 411008 India

**Keywords:** Plant biotechnology, Plant stress responses, Plant sciences, Biotechnology, Metabolomics

## Abstract

Cold-induced sweetening (CIS) causes considerable losses to the potato processing industry wherein the selection of potato genotypes using biochemical information has found to be advantageous. Here, ^1^H NMR spectroscopy was performed to identify metabolic perturbations from tubers of five potato cultivars (Atlantic, Frito Lay-1533, Kufri Jyoti, Kufri Pukhraj, and PU1) differing in their CIS ability and processing characteristics at harvest and after cold storage (4 °C). Thirty-nine water-soluble metabolites were detected wherein significantly affected metabolites after cold storage were categorized into sugars, sugar alcohols, amino acids, and organic acids. Multivariate statistical analysis indicated significant differences in the metabolic profiles among the potato cultivars. Pathway enrichment analysis revealed that carbohydrates, amino acids, and organic acids are the key players in CIS. Interestingly, one of the processing cultivars, FL-1533, exhibited a unique combination of metabolites represented by low levels of glucose, fructose, and asparagine accompanied by high citrate levels. Conversely, non-processing cultivars (Kufri Pukhraj and Kufri Jyoti) showed elevated glucose, fructose, and malate levels. Our results indicate that metabolites such as glucose, fructose, sucrose, asparagine, glutamine, citrate, malate, proline, 4-aminobutyrate can be potentially utilized for the prediction, selection, and development of potato cultivars for long-term storage, nutritional, as well as processing attributes.

## Introduction

Potato (*Solanum tuberosum* L.) – an important staple non-grain vegetable food crop – is used globally for both processing and table purposes. In order to ensure the year-round supply of quality tubers for consumption, cold storage of potato tubers after harvesting is mandatory to reduce sprouting, prevent diseases, avoid losses due to shrinkage, and extend post-harvest shelf life^1,2^. Usually 8–10 °C is the recommended storage temperature for potato tubers along with sprout suppressants, such as isopropyl *N*-(3-chlorophenyl) carbamate. The use of such chemical suppressants – due to their environmental safety and toxicity issues – are being replaced and reliance on storage of potatoes at colder temperatures even below 4 °C has been increased gradually^3–5^. During cold storage, potato tubers undergo cold-induced sweetening (CIS), wherein rapid degradation of starch and sucrose hydrolysis leads to the accumulation of reducing sugars (RS) – such as glucose and fructose^6^. During the frying process, these RS react with free amino acids in a Maillard reaction to generate dark-pigmented products that are bitter and unsightly to consumers. In addition to this, one of the products of the Maillard reaction is acrylamide – a potent neurotoxin and carcinogen^4,7^. Hence, CIS is considered as one of the critical parameters in potato production as well as in processing; therefore, the identification and development of potato tubers resistant to CIS has become a priority in a number of potato breeding programs^8–10^. Also, to meet the challenges of a frequently-changing market, production circumstances, and improving their economic condition^11^ as well as consumer’s preferences, it is crucial to develop potato cultivars that possess CIS resistance. In this regard, the metabolic stability of potato tubers during the cold storage period has been identified as one of the prime traits to be investigated for breeding programs worldwide^12^, wherein selection of potato genotypes at early generations, using biochemical information through marker-trait associations has been found to be advantageous^13^.

Metabolomic studies have been crucial in potato breeding mainly because tuber quality traits such as content and quality of starch, chipping quality, flesh color, taste, and glycoalkaloid content, etc., have been shown to be linked to a wide range of metabolites and alteration in metabolic networks^14–16^. Metabolic profiles in different life cycle stages of potato tubers were characterized to link temporal changes in metabolites to their acrylamide-forming potential^17^. In a recent study, comprehensive metabolomics and ionomics analysis on raw and cooked potato tubers of 60 unique genotypes spanning five marker classes were performed to understand the chemical variation and nutritional values in different varieties^15^. Such studies presented the metabolite diversity, which laid the foundation to generate potato cultivars with enhanced nutritional and processing qualities for human health.

Processing industry and consumers’ demand for potato cultivars with good CIS resistance, high specific gravity and dry matter (DM), low RS content^11,18^ along with improved nutritional qualities. In this regard, commercially grown processing (Atlantic and Frito Lay 1533) and non-processing (Kufri Jyoti and Kufri Pukhraj) potato cultivars^18^, are popular along with one locally grown table purpose cultivar (PU1) in India. While, Atlantic and Frito Lay-1533 have been rated as the good varieties for processing purpose with good storability^19^, Indian potato cultivars Kufri Pukhraj and Kufri Jyoti retain their suitability for table purpose due to their medium and average/poor storability, but have been found to be inferior for processing purposes due to high RS and low DM content^11,19–21^. Therefore, to advance our knowledge about the biochemical variation underlying CIS abilities of potato tubers, a comprehensive analysis needs to be performed from potato cultivars differing in their storability, as well processing attributes.

Here, we have carried out ^1^H nuclear magnetic resonance (NMR)-based untargeted metabolic profiling of five potato cultivars (mentioned above) differing in their CIS abilities and processing characteristics from freshly harvested (FH) potato tubers and after one-month of cold storage at 4 °C (CS). The key objective of this study was to examine the differences in metabolic profiles of these cultivars at harvest and after cold storage to further advance the knowledge of biochemical mechanisms underpinning the CIS trait. We report candidate metabolites (based on the metabolomics data and pathway analysis) that can potentially be used in breeding programs for the development of new cultivars with CIS resistance and improved processing attributes and thereby would enhance the potato tuber quality.

## Results and discussion

### Global profiling of metabolites in different potato cultivars – Processing versus non-processing cultivars

Global profiling of metabolites obtained from methanolic extracts of the FH and CS tubers obtained from five different potato cultivars, differing in their cold storage behaviour and processing characteristics^20–23^, identified a total of 39 abundant water-soluble metabolites using 1D ^1^H-NMR (Fig. S1). These were further confirmed using 2D ^1^H-^1^H TOCSY (Fig. S2) and information from the BMRB database. Water-insoluble metabolites in the organic phase gave broad and overlapping signals in 1D ^1^H-NMR and thus were excluded from the analysis.

**Figure 1:**
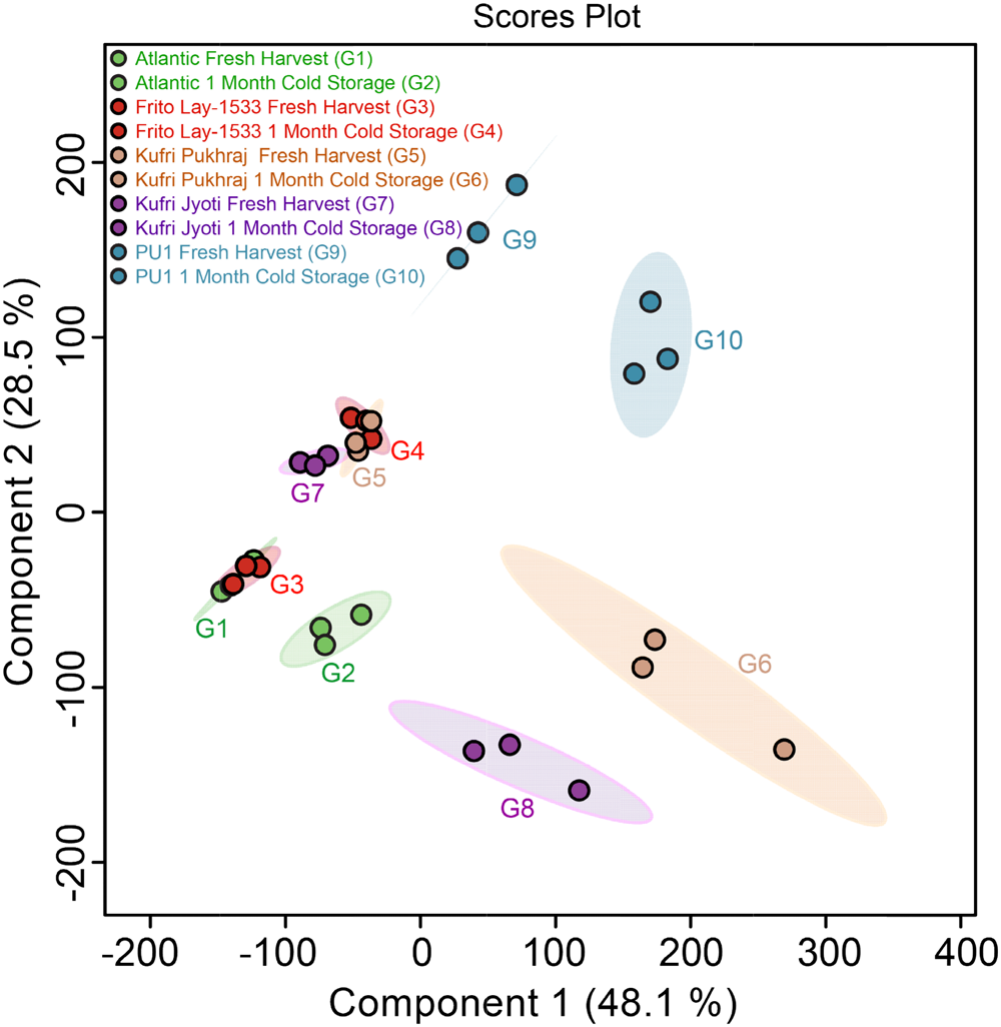
Scores plot as obtained by PLSDA utility of MetaboAnalyst 4.0 software for the different potato cultivars – Atlantic (G1, G2), Frito Lay-1533 (G3, G4), Kufri Pukhraj (G5, G6), Kufri Jyoti (G7, G8), and PU1 (G9, G10) at fresh harvest and one-month cold storage at 4°C. Three replicates were used for each cultivar and at each condition (as described in Methods). Ellipses showing 95% confidence limits of a normal distribution for each group of the samples have been marked in respective colours for each cultivar. Colour legends have been mentioned in the figure.

A range of distinct metabolites was detected that could be characterized mainly as sugars, sugar alcohols, amino acids, and organic acids (Table S2). The accumulation of these metabolites during various storage temperatures from different potato varieties have been previously reported^16,24,25^. Furthermore, PLS-DA analysis showed a divergent separation on the scores plot of PC1 and PC2, accounting for a 48.1% and 28.5% variation in the metabolites extracted from the FH and CS treated tubers of the different cultivars used in the study (Fig. 1). The unsupervised PCA plots for the different cultivars in the two conditions have been depicted in Fig. S3. Interestingly, the clustering of single points in the principal component space (marked by ellipses showing 95% confidence limits of a normal distribution) for metabolites from the FH tubers of Atlantic and FL-1533 (processing cultivars) clustered together while FH tubers from Kufri Jyoti and Kufri Pukhraj cultivars and the local PU1 were more similar to each other (Fig. 1). Further, the ellipses marking the principal component space for the metabolites of the cold storage tubers were also found to be different in processing, non-processing, and locally grown cultivars (Fig. 1).

In order to highlight the differences in the FH and the CS condition from the processing, the non-processing, and the local potato cultivars used in the study, pair-wise analysis was done (Fig. S4). PLS-DA analysis of metabolites obtained from the processing cultivars, Atlantic and FL-1533 showed 84.2% variation in PC1 and 10% variation in PC2 (Fig. S4A); and 84.4% variation in PC1 and 9.5% variation in PC2 (Fig. S4B), respectively. PLS-DA analysis of metabolites obtained from the non-processing cultivars, Kufri Jyoti and Kufri Pukhraj, showed 92.8% variation in PC1 and 3.8% variation in PC2 (Fig. S4C); 90.4% variation in PC1 and 6.1% variation in PC2 (Fig. S4D), respectively upon CS. Similarly, 92.9% variation in PC1 and 3.4% variation in PC2 (Fig. S4E) were observed for the metabolites obtained from the local cultivar PU1. Such differences due to metabolite content in the different cultivars at the two time-points could be attributed to the genetic make-up of each cultivar used in the present study. In fact, variability in the metabolite content has been reported in potato cultivars differing in their genetic background^26,27^. Using PCA, Dobson *et al*. showed the similarity and differences in genotypes belonging to different groups of potato on the basis of metabolic profiling^24^.

### Metabolic perturbations in processing, non-processing and local cultivars in CS conditions

**Figure 2:**
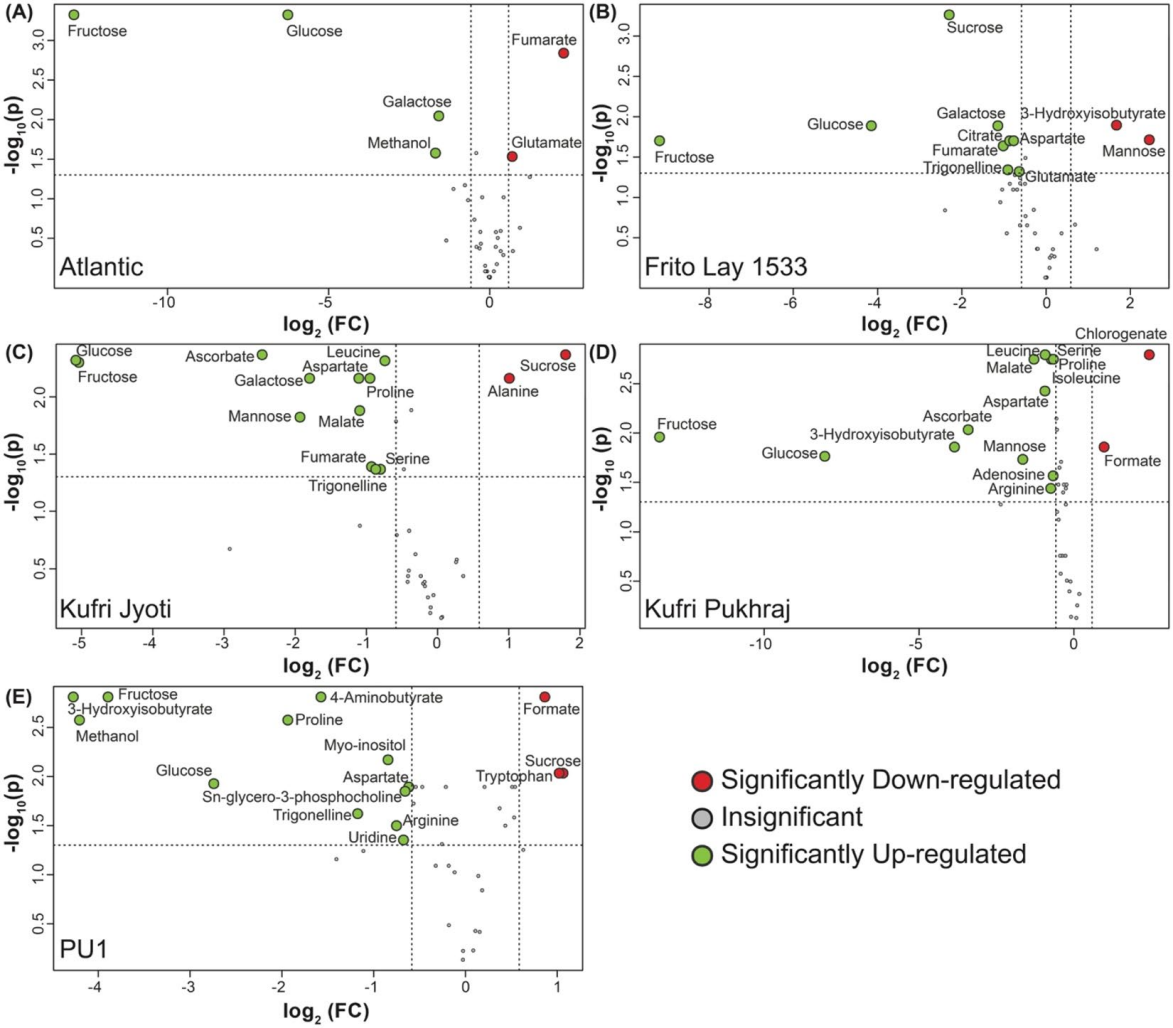
Volcano plots, where log_10_(FDR-corrected p-value) is plotted against log_2_(fold-change in concentration), depicting the changes in the metabolite concentration from freshly harvested potato tubers and tubers stored at 4°C for one month. The different cultivars used for the study have been depicted as A) Atlantic, B) Frito Lay-1533, C) Kufri Jyoti, D) Kufri Pukhraj, and E) PU1. The significantly down-regulated metabolites upon cold storage have been marked in red and the ones up-regulated have been marked in green.

**Figure 3:**
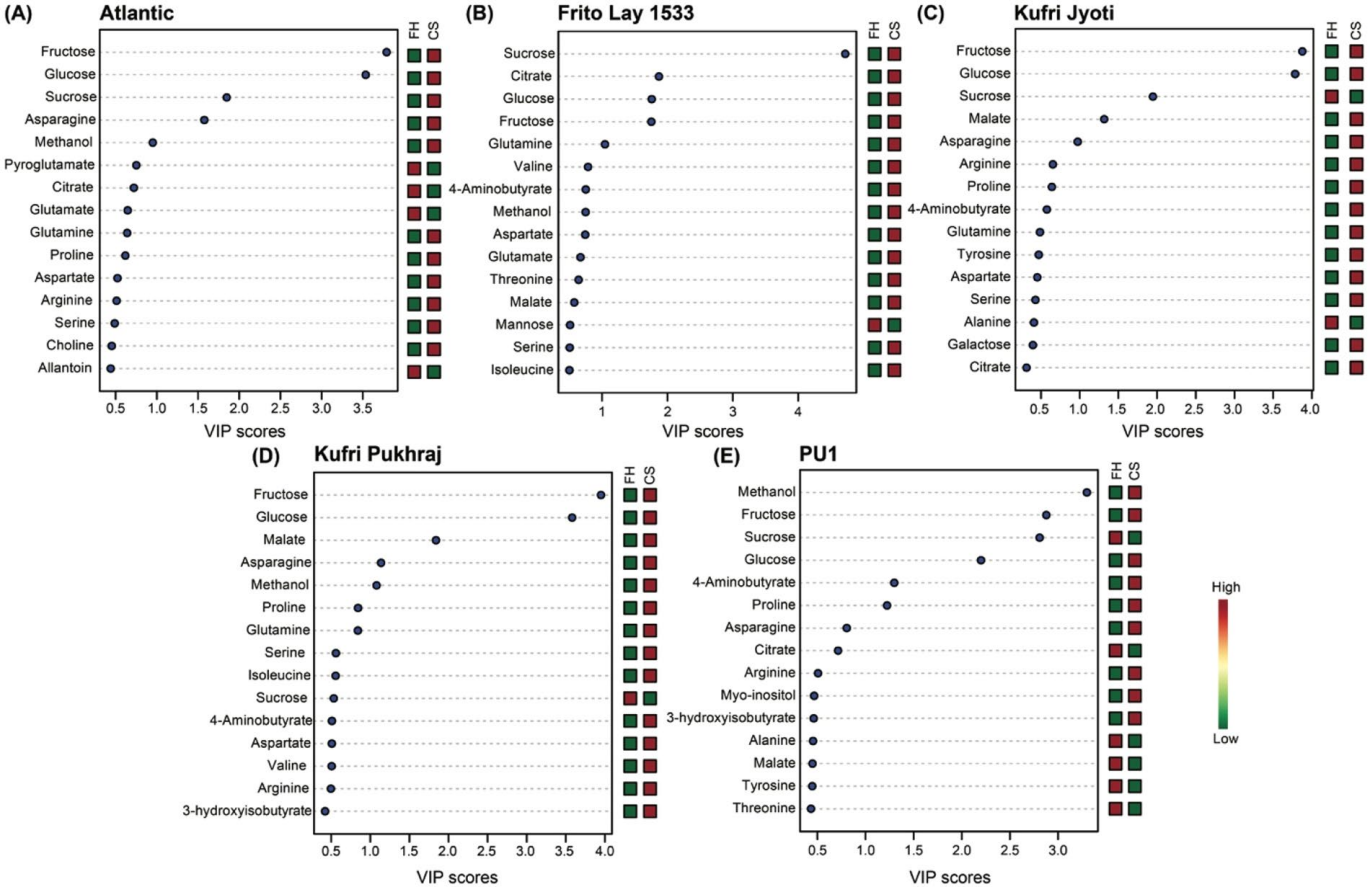
VIP scores obtained after pair-wise PLS-DA analysis for A) Atlantic, B) Frito Lay-1533, C) Kufri Jyoti, D) Kufri Pukhraj, and E) PU1. A VIP score of ≥1.0 is considered significant.

Volcano plot analysis (Fig. 2) and VIP score plot analysis (Fig. 3) were also performed under FH and CS conditions to identify the significantly affected metabolites in cold storage. Volcano plots help determine the importance and significance of the metabolite changes on the basis of p-value and fold change (FC) in concentration. On the other hand, VIP scores are used to discriminate significant metabolites on the basis of their PLS loading. Thus, a combination of the two is preferred to culminate key associated metabolites as there are least chances of losing any important metabolites of interest^28^.

Cold storage of potato tubers revealed that significantly altered metabolites varied with the cultivar studied. Nonetheless, the number of metabolites that significantly increased was higher than those that were decreased. Also, the number of metabolites that increased after cold storage was greater in Kufri Jyoti, Kufri Pukhraj, and PU1 cultivars as compared to Atlantic and FL-1533 (Fig. 2). These results indicate that one-month cold storage of potato tubers resulted in a gradual change of the metabolite content.

**Figure 4:**
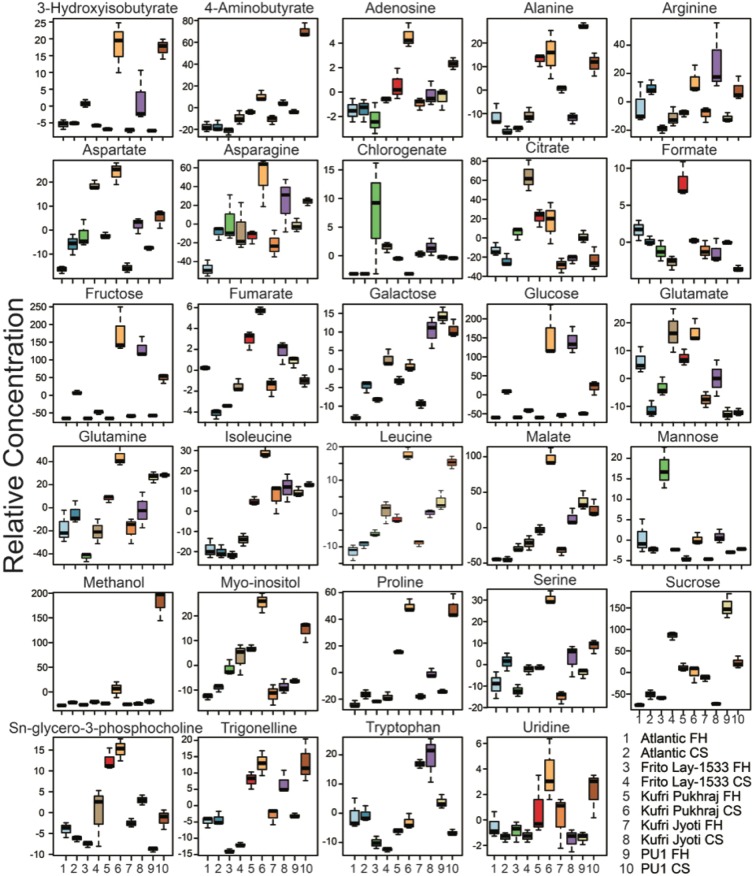
Box-Whisker plot for the significantly different metabolites (p-value <0.05, and with VIP score ≥1.0) for the different potato cultivars. The significantly different metabolites obtained from ANOVA and post-hoc analysis were selected individually and the relative concentrations of each of these were plotted against the two time-points, i.e., fresh harvest and one-month cold storage for the five cultivars used in the study. FH – fresh harvest and CS – cold storage at 4 °C.

Among all the cultivars, the processing cultivars i.e., Atlantic and FL-1533 were less reconfigured due to CS in contrast to non-processing cultivars (Kufri Jyoti, Kufri Pukhraj, and PU1). Glucose and fructose were more pronounced in the non-processing cultivars when compared to the processing cultivars (Figs. 2–4). While in the Atlantic cultivar, fumarate and glutamate were found to be significantly downregulated (*p* = 0.001; FC = 4.89 for fumarate and *p* = 0.029; FC = 1.62 for glutamate) upon CS when compared with FH (Figs. 2A and 3A); these metabolites showed significant upregulation (*p* = 0.022; FC = 0.49 for fumarate and *p* = 0.047; FC = 0.64 for glutamate) in FL-1533 upon CS (Figs. 2B and 3B). The opposite trend of these two metabolites in the processing verus non-processing cultivars indicates different adaptive strategies for the utilization of these metabolites upon CS, probably via the regulation of the expression of enzymes including, fumarase, aconitase, isocitrate dehydrogenase, glutamate synthase etc. Interestingly, asparagine was significantly increased in Kufri Jyoti (Figs. 2C and 3C) and Kufri Pukhraj (Figs. 2D and 3D) upon CS. The levels of organic acids, i.e., aspartate (*p* = 0.001; FC = 0.40), malate (*p* = 0.013; FC = 0.46) and fumarate (*p* = 0.040; FC = 0.52), were increased in the Kufri Jyoti cultivar upon CS treatment (Figs. 2C and 3C). Similarly, CS treatment of the Kufri Pukhraj (Figs. 2D and 3D) and the local PU1 (Figs. 2E and 3E) cultivar was associated with significant increase in malate (*p* = 0.001; FC = 0.40 for Kufri Pukhraj, respectively), aspartate (*p* = 0.003; FC = 0.52 for Kufri Pukhraj and *p* = 0.012; FC = 0.65 for PU1) and methanol (*p* = .002; FC = 0.05 for PU1).

Cold storage has been known to induce modifications in plants metabolome^29,30^. The effect of cold storage on metabolite levels in different potato cultivars can be correlated with the differential enzyme activities or cold signalling and altered gene expression patterns. For instance, the differential metabolite levels observed after cold treatment in six peach fruits were reflected as transcriptional and/or posttranscriptional responses^29^. Integration of metabolomics with quantitative genetics found an effective approach to identify the candidate genes underlying metabolite variations which offers trait-specific markers to improve commercially important traits^31^. CIS trait is expected to be associated with primary metabolites and an untargeted metabolic quantitative trait loci (QTL) analyses has revealed a relationship between primary metabolism and potato tuber quality^14^. Metabolic profiling performed for 26 starch and CIS-related traits on 97 potato genotypes using gas chromatography-time of flight-mass spectrometry detected a total of 139 polar metabolites. Of these 139, metabolite quantitative trait loci were identified for approximately 72% of the detected compounds. In particular, between different amino acids, the QTL for starch phosphorylation was found to be co-localized with mQTLs for alanine, GABA, pyroglutamate, phenylalanine, serine, threonine, aspartic acid, glutamine, tyrosine and tryptophan^14^. Thus, using metabolomics, the key metabolic pathways and the key candidate genes affecting a trait can be identified in plants. These genes can be further manipulated using genetic engineering approaches to alter their function^32^. Therefore, we propose that the metabolite variability obtained in the present study can be combined with the genetic information that will facilitate the discovery of metabolite biomarkers for CIS and potato processing related traits in the cultivars used in the study.

### Metabolic correlation network analysis

Metabolite correlations of potato groups differing in the genetic background have been previously reported^24,33^. In the current study, Pearson’s correlation coefficient analysis was used to analyse the metabolite-metabolite correlation among identified metabolites in all five cultivars at both the time-points (Figs. S5–S7). Correlations within a cultivar have been plotted with an aim to identify correlations between metabolites in a given treatment. Two correlation plots have been plotted in each half of the square (separated by the diagonal) to highlight and compare the differences in metabolite-metabolite correlations within FH with that from CS conditions. The aim was to map all the significant metabolite correlations in both the treatments for each cultivar. A total of 100, 84, and 45 significant correlations (p-value < 0.05) were obtained at FH among processing group (Atlantic and FL-1533), non-processing group (Kufri Pukhraj and Jyoti), and local (PU1) cultivars, respectively (upper-right half of the plot marked with white triangle in Figs. S5–S9). After CS, the number of significant correlations changed to 108, 105 and 31 (lower-left half of the plot marked with blue triangle in Figs. S5–S9) in the processing group, non-processing group, and local cultivar, respectively. The number of positive vs. negative correlations also varied depending on the variety (Table S3). Remarkably, among all the metabolites, amino acids dominated the significant metabolite correlations. In general, the metabolite-metabolite correlations detected in the present work were highly dependent on the type of the cultivar considered; however, some particular behaviours of the metabolic network after CS are worth mentioning. For instance, a positive correlation of fructose with phenylalanine, valine, 4-aminobutyrate, glutamine, choline, and glutamate was evident in Atlantic (Fig. S5) upon CS. In the case of FL-1533, pyroglutamate was found to be positively correlated with valine, alanine, arginine, and 4-aminobutyrate after CS (Fig. S6). Whereas 4-amiobutyrate was positively correlated with trigonelline, sucrose, allantoin, and arginine; citrate and malate displayed significantly positive correlations with several other metabolites in Kufri Jyoti after CS (Fig. S7) which were not evident in the other cultivars. However, several negative correlations were exclusively observed in PU1 cultivar after CS (Fig. S9). Glutamate and glutamine are metabolic neighbours in the glutamine synthase pathway and show no correlation in the non-processing and local cultivars (Figs. S7–S9) in FH as well as CS treatments, while are found to be correlated in processing cultivars (Figs. S5 and S6). Significant correlations among various potato cultivars might help to predict the CIS status of the particular potato genotype based on FH and CS tuber profiling. However, the reason for these strong correlations remains unclear as no direct link has been reported so far and further investigations are warranted.

Significant metabolite variation and metabolite-metabolite correlations were detected from a collection of 60 unique potato genotypes that span 5 different market classes such as russet, red, yellow, chip, and speciality^33^, where authors concluded that metabolite diversity and correlations data can support the potential to breed new cultivars for improved health traits. The metabolite variations (Fig. 4) and various correlations (Figs. S5–S9) obtained especially after cold storage raise the possibility to test the function of genes or the combination of transgenes using genetic engineering approaches to further validate the role of other candidate genes identified in this study.

### Metabolic pathway analysis and potential metabolite insights of CIS

**Figure 5:**
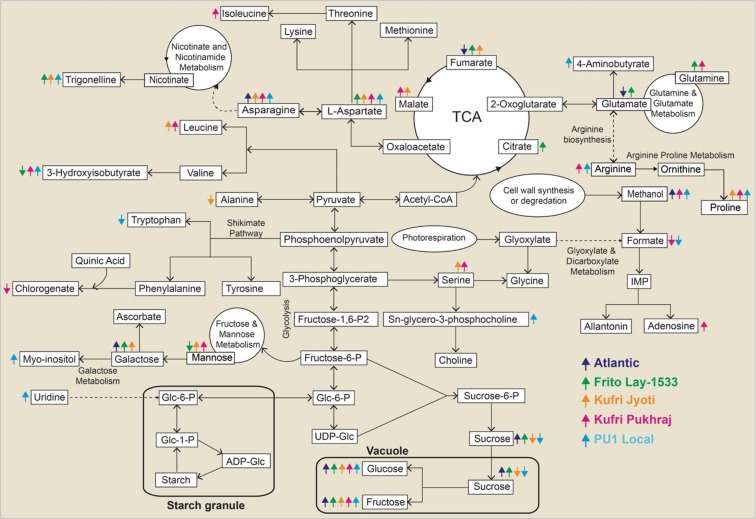
Pictorial representation of metabolic pathways affected during cold-induced sweetening in the different cultivars of potato. The significantly different metabolites under cold storage have been marked with arrows wherein an ↑ indicates upregulated metabolites and the ↓ arrow indicates down regulated metabolites. TCA – tricarboxylic acid.

CIS is a multigenic complex trait involving multiple intricate metabolic pathways which clearly indicates that it is unlikely to be controlled by a single metabolite; thus, multiple metabolites would come-up as plausible candidates for CIS in potatoes. We performed the pathway analysis depicting significantly affected metabolites in cold-stored potato tubers by comparing the primary metabolites based on KEGG and the reference pathway^4,34^ (Fig. 5). Potato tubers displayed diverse biochemical mechanisms during CIS and the amount of sugar in potato tubers is influenced by several candidate genes operating in glycolysis, hexogenesis, and mitochondrial respiration^4,35^. The metabolic pathway analysis presented in this study suggests that several metabolites were affected during cold storage and mainly resulted from the alanine, aspartate, and glutamate metabolism; valine, leucine, and isoleucine biosynthesis; arginine and proline metabolism; glycine, serine, and threonine metabolism; the TCA cycle, fructose and mannose metabolism, galactose metabolism, nicotinate and nicotinamide metabolism, glycolysis; and starch and sucrose metabolism along with several other metabolites (Fig. 5). Also, the levels of metabolites were found to be specifically different depending on potato cultivars (Figs. 2–4) indicating that the specific metabolites might play a crucial role in determining the cold-induced ability of potato cultivars. Also, the molecular events controlling such metabolic perturbations in potato tubers after cold storage are still puzzling.

### Sugar metabolism under cold storage condition

Cold temperature induces starch degradation in potato tubers to form sucrose, glucose, and fructose, thereby leading to an imbalance between starch degradation and sucrose metabolism in tubers. The enzymes involved in these processes such as starch synthases, invertase, invertase inhibitors, sucrose synthase, UDP-glucose pyrophosphorylase, etc., have been extensively studied at biochemical and molecular levels^4,12,35–40^. Storage of potato tubers at 4 °C significantly increased the levels of sucrose, particularly in Atlantic (VIP = 1.82) and Frito Lay 1533 (p-value <0.001; FC = 0.20), while it was significantly decreased in Kufri Jyoti (*p* = .004; FC = 3.47) and PU1 (*p* = .009; FC = 2.08), and remained invariant in Kufri Pukhraj (Fig. 4). On the other hand, the increase in RS was more pronounced in the non-processing cultivars – Kufri Pukhraj, Kufri Jyoti, and PU1; as compared to the processing cultivars – Atlantic and Frito Lay 1533 (Figs. 2–4). These results are coherent with previous studies that observed an increase in RS after cold storage of potato tubers^11,21,23,38^, which has been attributed to the enhanced activity of the vacuolar invertase^33^. The accumulation of RS in tubers of cold-sweetening susceptible and cold-sweetening resistant potato cultivars was found to be under the control of vacuolar invertase and invertase inhibitors^12,38,41^. The levels of RS were decreased after one month of cold storage and this was accompanied with increased expression of the vacuolar invertase inhibitor mRNA accumulation in cold-sweetening resistant cultivars. Therefore, a relatively lower increase of RS after cold storage in Atlantic and Frito Lay 1533 cultivars (when compared with non-processing and local cultivar) used in this study could be attributed to increased levels of vacuolar invertase inhibitor. Allelic variations in the vacuolar invertase inhibitor gene from Atlantic, Frito Lay 1533, Kufri Jyoti, Kufri Pukhraj, and PU1 cultivars could be associated with the variation in RS levels in these cultivars^38^. However, these results need to be further validated using a qRT-PCR expression of vacuolar invertase inhibitor gene before and after cold storage in these cultivars. In addition to this, FL-1533 is represented by higher dry matter and starch content as compared to the non-processing cultivars^11^. However, further biochemical experiments and transcriptomics data would be needed to comment on the set of enzymes that are being upregulated or downregulated in specific cultivars thereby changing the metabolome upon CS.

### Amino acid metabolism under cold storage condition

The accumulation of amino acids has been reported in cold stress and has been found to improve resistance to cold temperature in several plant species^42,43^. Metabolic correlation network analysis revealed (Figs. S5 to S9) that the amino acids dominated the significant metabolite correlations indicating an important role CIS process of potato tubers. In the amino acid metabolism pathways, the distinct significantly affected pathways include metabolism of 11 amino acids: isoleucine, glutamate, glutamine, leucine, alanine, arginine, proline, tryptophan, aspartate, asparagine and serine metabolism. In the present work, although proline levels were increased in all the potato cultivars upon cold storage; tubers of non-processing cultivars (Kufri Jyoti, Kufri Pukhraj, and PU1) exhibited relatively higher levels of it (Fig. 4). The GABA shunt pathway was significantly enhanced as seen by the increased levels of 4-aminobutyarte in PU1 (*p* = .001; FC = 0.33) upon cold storage, whereas it was not significantly affected in any other potato cultivar. Accumulation of proline and GABA has been reported in plants under chilling stress^44,45^ wherein these were presumed to act as osmolytes (Figs. 4 and 5), as an acclimation response under CS treatment^46^. GABA treatment has been demonstrated as a promising approach not only for reducing the enzymatic browning but also maintaining the quality of fresh-cut potatoes^47^. However, to date there has been no conclusive evidence on the role of several of these metabolites in CIS process of potato tubers. This is particularly because the comprehensive analysis of the biochemical pathways affected during CIS remains to be elucidated.

It is noticeable that the levels of glutamine were exclusively significantly higher in FL-1533 and Kufri Pukhraj after CS as compared to the rest of the potato cultivars (Figs. 4 and 5) probably due to an elevated transcription of glutamine synthetase^48^. Enzymes, branched-chain amino acid aminotransferase and glutamine synthetase, involved in glutamine biosynthesis have been found to be potentially involved in governing potato tuber quality traits^49^. The changes in the levels of total amino acids, specifically the levels of asparagine, and the ratio of free asparagine to RS during cold storage were found to be significantly varied among different potato cultivars upon CS (Fig. 4). These factors, therefore, can further influence the processing quality of potato tubers.

In addition to the amount of RS, the total and individual amino acid content, the asparagine content, levels of organic acids, and other metabolites have been considered as important processing parameters^50^. Breeders aim for the identification and development of processing potato cultivars with low free-asparagine and RS as desirable characteristics for processing purpose. In the current study, a unique metabolite combination was observed for the processing cultivar, FL-1533, which was represented by the lowest amount of RS and asparagine compared to rest of the cultivars CS (Figs. 2–4).

### TCA cycle metabolites under cold storage condition

Organic acids are known to play an important role in cold tolerance in plants^45,51^. Amongst the different TCA cycle metabolites, the levels of citrate were significantly higher (*p* = 0.019; FC = 0.54) in the processing cultivar, FL-1533; whereas Kufri Jyoti and Kufri Pukhraj showed significantly higher levels of malate after CS (Fig. 5). Low temperature has been shown to increase citrate levels in banana, tomato and Ponkan fruits thereby indicating that citrate might be involved in cold tolerance of these fruits^45,51,52^. Increased levels of citrate upon cold storage at 4 °C for 28 days in tomato fruits have been attributed to the enhanced expression ATP-citrate synthase and isocitrate dehydrogenase^51^. Cold storage resulted in an increase in different isoforms of malate dehydrogenase, isocitrate dehydrogenase and 2-oxoglutarate dehydrogenase genes in cold-stored (4 °C) litchi fruit which accelerated the fruit senescence that might be associated with cold susceptibility or respiratory burst^53^. In potato, both citrate and malate are critical in the determination of non-enzymic browning reactions, the suitability of potato tubers for culinary use, industrial processing, the texture of cooked and fried potato products, after cooking darkening and physiological age/ stages of development in the storage of potato tubers^54–58^. Significantly higher citrate levels in FL-1533 (*p* = 0.019; FC = 0.54) exhibited as compared to rest of the cultivars after CS (Fig. 4), might be associated with CIS resistance with good processing attributes as citric acid is known as a popular anti-browning agent, mainly because it not only inhibits the polyphenol oxidase by reducing pH but also chelates copper at the enzyme-active site^59^. Hence, it is necessary to develop the indicators of tuber browning and physiological age, mainly because both the growers and seed companies can optimize the storage conditions for individual cultivars^56^. A molecular-function map for carbohydrate metabolism and transport has mapped both citrate synthase and malate dehydrogenase on potato chromosomes^37,60,61^. So far, no functional markers have been developed for these candidates. Therefore, potato cultivars differencing in their CIS ability could be further screened for the quantitative variations in citrate and malate levels. Such data then can be utilized for the development of molecular-linkage maps based on functional gene markers (molecular-function maps) which are necessary for a candidate-gene approach to further identify genes responsible for quantitative traits at the molecular level^37^.

Importantly, several other metabolites such as fumarate, adenosine, sn-glycero-3-phosphocholine, 4-aminobutyrate, 3-hydroxyisobutyrate, trigonelline, and chlorogenate were significantly affected upon CS (Figs. 2, 3 and 5) thereby implying the role of these as candidate metabolites in the CIS process. Interestingly, methanol levels were significantly higher in PU1 cultivar (Figs. 2E and 3E). The texture of potato tubers is mainly determined by starch properties, cell size, cell-wall structure and composition, the breakdown of the cell wall middle lamella during cooking, and the correlation between pectin methyl esterase activity and the degree of methylation of cell wall pectin^62^. The amount of methanol released on saponification is the measure of the degree of pectin methylation and found indirectly associated with the potato tuber texture properties^62^. Therefore, the amount of methanol present in potato tubers can be used as a potential marker for screening of potato cultivars for texture properties. However, the possibility cannot be ruled out that since methanolic extracts were used for the metabolomics analysis; therefore, it is likely that, the methanol detected by NMR might represent the exogenous levels.

Likewise, myo-inositol was significantly upregulated in PU1 cultivar (*p* = 0.006; FC = 0.55), indicating its role in cold tolerance^63^. However, further studies are necessary to understand the role of myo-inositol in CIS process of potato tubers. The convergence and divergence of various pathways involved in CIS revealed a complex metabolic network. However, the roles of these metabolites and their accumulation pattern in response to cold storage in different potato cultivars can be further investigated by identification of candidate genes which are putatively involved in the formation of enzymes involved in the biosynthesis of these metabolites. In order to improve potato (*Solanum tuberosum* L.) genotypes through selection or breeding, it is helpful to determine the chemical composition of tubers^64^. Previous studies have suggested that various primary metabolites in potato tubers can be utilized as biomarkers in breeding programs for predicting agronomically important traits such as black spot bruising and chip quality^65,66^.

Maintaining the quality of potato tubers during storage is a major challenge. Therefore, the biochemical information on the response of potato cultivars to cold storage and metabolite accumulation can be useful for the development of candidate metabolites for predicting the severity of CIS of different potato genotypes. Such candidates can then be tested on a wide range of potato genotypes differing in CIS response and the information obtained can be easily integrated into the existing potato storage management and breeding methods. Moreover, such predictive candidate metabolites can be used in selection for potato breeding and for tailoring storage conditions for the harvested tubers^67,68^. Furthermore, candidate metabolites can be utilized for the manipulation of a specific metabolite pathway for developing potato genotypes with improved processing characteristics.

## Conclusions

A number of commercial potato cultivars used for processing and table purpose are currently available. However, the information on biochemical variations underlying the CIS status of various potato cultivars is very scanty. Our studies showed that the differences in variation in several tuber metabolite contents such as glucose, fructose, sucrose, asparagine, glutamine, citrate, malate, proline, 4-aminobutyarte, etc., especially after cold storage which indicated that these candidate metabolites could be used to unravel the biological basis of CIS process of potato tubers. For instance, low levels of glucose, fructose, and asparagine accompanied by high citrate levels in processing cultivar (FL-1533) and higher glucose, fructose and malate levels in non-processing cultivars (Kufri Pukhraj and Kufri Jyoti) can be utilized for the prediction, selection and development of potato cultivars for altered storage as well processing attributes. Likewise, variations in GABA and glutamine can be utilized in determining the enzymatic browning of fresh-cut potatoes and tuber quality respectively. The correlation between the amino acids, organic acids and sugars suggests for the close biochemical relationship as well the interdependence of the amino acid metabolism, the tricarboxylic acid (TCA) cycle, and glycolysis in CIS process of potato tubers. Both proline and malate were found to be correlated in Kufri Jyoti and Kufri Pukhraj after cold storage, indicating that these metabolites can be useful in the identification of CIS susceptible potato cultivars. Also, the presence or absence of specific metabolites cannot be the only concluding answer for the prediction of CIS behaviour. The relative amount of the specific metabolite present can also play a contribution in CIS status of potato cultivar. Follow-up biochemical studies in the cultivars reported, in addition to metabolomics experiments from another 30–40 additional potato cultivars from India, with longer storage conditions (2–4 months, in addition to one month) would be required in future to ascertain the results obtained in the study in the future. Such information on metabolic biomarkers would be of major interest to potato breeders and the processing industry in the selection of CIS resistant potato genotypes.

## Methods

### Plant Material

Readily available exotic processing potato cultivars – Atlantic and Frito Lay-1533 (FL-1533) (Pepsi Foods Pvt. Ltd. Channo, Sangrur), and two Indian non-processing/table purpose cultivars – Kufri Jyoti and Kufri Pukhraj (Central Potato Research Institute, Shimla)^11,20–23^, along with a locally grown cultivar – PU1, were used in the present study. These varieties were obtained from BT Company and Jai Kisan Farm Products and Cold Chains Pvt. Ltd, India, Pune. These cultivars have also been found to differ in their CIS ability^19,22,23^. The locally grown potato cultivar – (PU1) – used for table purpose, has been reported to contain high RS after cold storage^38^, and possesses poor storability in cold temperatures. The information on the physiology of potato cultivars has been presented in Supplementary Table S1.

### Potato plantation and harvesting

The tubers from 5 potato cultivars – namely, Atlantic, FL-1533, Kufri Pukhraj, Kufri Jyoti, and PU1 – were planted under open-door conditions (natural conditions and sunlight) in triplicates in PB5 Polythene bags containing potting mix (50% shredded pine bark, 20% crusher dust, 10% cow dung, 20% soil supplemented with sand and slow-release fertiliser) on 20^th^ November 2017, at the Department of Biotechnology, Savitribai Phule Pune University, Pune, India. Field facility was not available and the experiment was conducted as a randomized potted trial. The plants were regularly watered. Tubers were then harvested on 5^th^ March 2018 after full senescence. Six tubers (medium to large sized) from three replications (two tubers from each replicate) of each potato cultivar were transferred to a paper bag. The remainder of the harvested tubers from each cultivar were used for propagation/maintenance purposes. The tubers were washed under running tap water, cored and de-skinned. Of the six harvested tubers, three tubers (one tuber from each replication) were immediately processed for the fresh analysis (fresh harvest; FH) and the other three tubers (one tuber from each replication) were stored in the dark for one month at 4 °C in paper bags (cold storage; CS) for extraction of metabolites. All the tuber samples were subjected to freeze-drying (Operon, FDB-5503, Korea) for one week before using for metabolite extraction.

### Metabolite extraction

The freeze-dried potato tuber samples were ground to a fine powder and were used for metabolite extraction. Briefly, approximately 200 mg of freeze-dried potato powder was re-suspended in 200 µl Phosphate Buffer Saline (PBS) in 1.5 ml tubes and vortexed for five minutes. To each tube, 400 µl ice-cold methanol (Sigma, HPLC grade) was added, followed by vortexing for another 5 min. Samples were then stored at −20°C for 12 h. Post-incubation, the samples were centrifuged at 16,000 ×*g* (Eppendorf centrifuge 5415 C, Hamburg, Germany) for 20 min at 4 °C. The supernatants were transferred to fresh 1.5 ml Eppendorf tubes and were subjected to lyophilization (Operon, FDB-5503, Korea). There were three replicates for each of the five cultivars processed in two treatments (a- FH; and b- CS) making a total of 30 distinct samples. The lyophilized extracts of all the samples were reconstituted into 580 µl 100% NMR buffer (20 mM sodium phosphate, pH 7.4 in D_2_O containing 0.4 mM DSS (2,2-dimethyl-2-silapentane-5-sulfonic acid). For making buffer containing a known concentration of DSS, 17.46 ± 0.01 mg of DSS was weighed (mol wt. 218.32 g/mol) and dissolved in 2000 µl ± 2 µl of phosphate buffer. This stock solution was then diluted to 100-fold resulting in a final buffer solution containing 87.30 ± 0.16 mg L^−1^ of DSS in solution, which corresponds to 399.9 ± 0.7 µM of DSS in the buffer. The samples were vortexed for 2 min at room temperature and centrifuged at 4000 ×*g* for 2 min. The supernatants were transferred to respective 5 mm NMR tubes for NMR data measurements.

### NMR spectroscopy

All the NMR data was measured on a Bruker AVANCE III HD Ascend NMR spectrometer operating at 14.1 Tesla. This spectrometer has been equipped with pulsed-field gradients in x, y, and z directions (operating at 54 Gauss/cm), and Bruker high-performance shim system with 36 orthogonal shim gradients and integrated real-time shim gradient for 3-axis shimming. A cryogenically cooled quad-channel (^1^H/^13^C/^15^N/^31^P-^2^H) probe was used to pump radio frequencies and detection. All the NMR data was measured at 298 K controlled by the Bruker VT unit. NMR data measurement, metabolite identification, and quantification have been reported in details as per Metabolomics Standards Initiative (MSI) as described recently by our group^69,70^. Water-suppression pulse sequence from Bruker library (noesygppr1d) was used to measure all the ^1^H NMR data, where water suppression was achieved by pre-saturating water using continuous-wave irradiation at 5.56E-05 W during the inter-scan relaxation delay of 5 s, and employing spoiler gradients (Smoothed square shape SMSQ10.100, where G_1_ was with 50% power and G_2_ was with -10% power for 1 ms duration each). The data acquisition period of 6.95 s (including inter-scan delay) was used, giving a spectral width of 7200 Hz resolved in 32k data points. Sixty-four scans were used to average the signal recorded on each sample. ^1^H 90° pulse-width, receiver gain, and water-suppression parameters were kept invariant from sample to sample to rule out intensity variations while recording data on different samples. To help with assignment of metabolites, ^1^H-^1^H total correlation spectroscopy (TOCSY) experiment (using ‘mlevesgppg’ pulse sequence from Bruker library) was measured with a 6000 Hz of spectral width resolved in 2048 × 1024 data points with 40 transients per increment. A Hartman-Hahn mixing time of 80 ms was employed for the TOCSY spin-lock using composite blocks of 90°-180°-90° pulses with 90° pulse width of 25 μs at 2.29 W of power. TOCSY data was recorded in States-TPPI mode and Smoothed square shaped (SMSQ10.100) gradients were used with 31% power (after the spin-lock period) and 11% power (before refocusing) for a duration of 1 ms.

### Metabolite identification and quantification

All of the NMR data were processed using Topspin (v3.5) software (www.bruker.com/bruker/topspin). ^1^H NMR raw data was multiplied with exponential function and zero-filled to 64 K data points prior to Fourier transformation. All the spectra are manually phased and the baseline is corrected before subjecting to further analysis. ^1^H chemical shift was directly referenced to DSS resonance (δ=0 ppm at 25 °C). ^1^H-^1^H TOCSY was processed with a pure cosine function (SINE with SSB = 2) and zero-filled to 2048 and 1024 data points in F1 and F2 dimensions prior to subjecting the data to Fourier transformation. Multiple peak parameters including, chemical shift values, J-coupling values, line shape, and multiplicity information, in combination with Biological Magnetic Resonance Bank (BMRB) and Human Metabolome Database (HMDB) were used to assign the peaks to respective metabolites. Chenomx NMR suite 8.1 (https://www.chemnomx.com/) was used to carry out the ^1^H resonance assignment with a chemical shift tolerance of 0.05 ppm when comparing the data with BMRB/HMDB. Resonance assignment of metabolites was confirmed using ^1^H-^1^H TOCSY (Fig. S1) cross peak pattern of individual metabolites containing coupled ^1^H spin systems via a semi-automated software, MetaboMiner (http://wishart.biology.ualberta.ca/metabominer/). A set of five resonances remained unassigned and have been duly marked as U1-U5 (Fig. S2 and Table S2).

After identification of metabolites, respective peaks were manually picked, integrated using Topspin v3.5, and converted to absolute concentrations of individual metabolites using Chenomx v8.1 by comparing with the peak integrals from an external reference compound DSS of known concentration (400 µM). The absolute concentrations obtained above were then normalized using the dry weight obtained from the tuber mass used for metabolite extraction. The data matrix file was created using concentrations of metabolites as obtained above from 30 distinct samples. The lower limit of quantification achieved using the above-mentioned NMR parameters was 0.25 μM for the methyl peak of DSS at a s/n ratio of 10.

### Metabolic pathway analysis

Metabolic pathway analysis depicting significantly affected metabolites in cold-stored potato tubers was performed by comparing the primary metabolites based on KEGG and the reference pathway^4,34^, using MetaboAnalyst 4.0 web tool (https://www.metaboanalyst.ca/).

### Statistical analysis

Due to high dimensionality and large complexity (5 cultivars in triplicates in 2 processing conditions with each NMR sample having ~1000 ^1^H signals) of the metabolomics data, multivariate statistical analysis was carried out to depict the differences in concentrations of metabolite in various cultivars upon CS. Principal Component Analysis (PCA) and Partial Least Squares Discriminant Analysis (PLS-DA) were carried out using normalized metabolite concentration as input in the MetaboAnalyst 4.0 (www.metaboanalyst.ca) to provide insights into the separations between the FH and CS tubers in all five cultivars studied. PCA is a technique that transforms the variables in a data set into a smaller number of new latent variables called principal components (PCs), which are uncorrelated with each other and account for decreasing proportions of the total variance of the original variables. On the other hand, PLS-DA, a supervised extension of PCA is used to maximize the covariance between predictor variables (metabolite concentration from NMR measurement) and the response variables (e.g. the classes of each sample). PCA was applied to identify the clustering patterns while as PLS-DA was used for classification and feature selection in the metabolic dataset. The input data table was normalized using the Pareto-scaling approach available in the MetaboAnalyst prior to multivariate analysis. Pareto-scaling was chosen over the unit variance scaling approach as it gives more importance to abundant metabolites thereby avoiding noise from low signal-to-noise peaks into the analysis. Pareto-scaling has typically been the method of choice in NMR-based metabolomics studies^71–73^. Correlation between the first two principal components was drawn as the scores plot for all the samples and clusters of normal distribution were marked using ellipses showing 95% confidence limits for each group in PCA analysis. The PCA and PLS-DA score plots for all five cultivars showed that the clusters of FH and CS treatment tubers are well separated from each other thereby indicating different metabolic profiles. Next, pair-wise analysis of all five cultivars in FH and CS treatments was achieved using volcano plot analysis, where metabolites were selected based on dual criteria: 1) the significance (false discovery rate (FDR) corrected p-value < 0.05); and fold-change in concentration (cut-off for fold change was set to 1.5 fold increase or decrease). In addition to this, the variable importance of projection (VIP) score plot obtained by PLS-DA identified the key metabolites responsible for the clustering of various groups. VIP score measures the contribution of a variable to the PLS-DA model. Metabolites with a VIP score of ≥1.0 are generally considered to be statistically significant^74,75^. A union set of significant metabolites (those identified from volcano plot analysis, and from VIP score following the above-mentioned criteria) were taken for generating box and whisker plots to highlight the variation of a particular metabolite across replicates, in different cultivars, and in different treatment conditions. Metabolites, e.g., ascorbate, having low signal-to-noise (s/n < 15) in NMR spectra, although identified with confidence, were not included in box and whisker plot analysis as they might be prone to over- or under-estimation of concentrations. Further, Pearson’s correlation was used to generate the correlation plots to identify all the significantly correlated metabolites (p-value < 0.05) in FH and CS treatments for all five cultivars. The correlations between FH and CS treatment for each cultivar was drawn by combining the data for three replicates. The significantly affected metabolic pathways were then identified using significantly perturbed metabolites as input in MetaboAnalyst tool and KEGG pathway database (www.genome.jp/kegg/pathway.html).

### Ethical approval

This article does not contain any studies with human and/or animal participants performed by any of the authors.

## Supplementary information

Supplemental Information.
