# Supplementary material for: Cold storage reveals distinct metabolic perturbations in processing and non-processing cultivars of potato (Solanum tuberosum L.)
Source: Sci Rep. 2020 Apr 14;10:6268. doi: 10.1038/s41598-020-63329-5 (PMC7156394; doi:10.1038/s41598-020-63329-5)

Title: Cold storage reveals distinct metabolic perturbations in processing and non-processing cultivars of potato (*Solanum tuberosum* L.)

Author names: Sagar S. Datir[a,b,1,*], Saleem Yousf[c,1], Shilpy Sharma[a] , Mohit Kochle[a], Ameeta Ravikumar[d], and Jeetender Chugh[c,e,*]

Author address:

[a]Department of Biotechnology, Savitribai Phule Pune University, Ganeshkhind, Pune 411007, India

[b]Present address: Biology Department, Biosciences Complex, Queen's University, Kingston, ON, CA K7L 3N6.

[c]Department of Chemistry, and [e]Department of Biology, Indian Institute of Science Education and Research, Pune 411008, India

[d]Institute of Bioinformatics and Biotechnology, Savitribai Phule Pune University, Ganeshkhind, Pune 411007, India

[1]Authors contributed equally

[*]**Corresponding authors:**

Sagar S Datir, Ph.D.

Address: Department of Biotechnology, Savitribai Phule Pune University, Ganeshkhind, Pune 411007, India

E-mail: sd153@queensu.ca, Phone: +918412013810, ORCID: 000-0003-0065-498X

and

Jeetender Chugh, Ph.D.

Assistant Professor, Department of Chemistry & Biology,

C-115, Indian Institute of Science Education & Research, Dr. Homi Bhabha Road, Pashan, Pune 411008, India

E-mail: cjeet@iiserpune.ac.in, Phone: +91-20-25908121, +91-8378979667, ORCID: 0000-0002-9996-5202

Supplementary Table S1

Information on potato cultivars

Variety / Cultivar	Source	Type	Shape	Colour		Storage behaviour
				Skin	Flesh	
Frito Lay-1533	Pepsi Foods Pvt. Ltd. Channo, Sangrur	Processing	Oval	Light russet	White	Good
Atlantic	Pepsi Foods Pvt. Ltd. Channo, Sangrur	Processing	Round	Brownish yellow	White	Good
Kurfi Pushkraj	Central Potato Research Institute, Shimla	Non-processing/Table purpose	Oval	Brown	Cream	Average
Kurfi Joyti	Central Potato Research Institute, Shimla	Non-processing/ Table purpose	Round	Brownish yellow	Cream	Medium
PU1	No information	Non-processing/ Table purpose	Oval	Brownish yellow	Cream	Medium

The information is adapted from, Kaur and Aggarwal, 2014; Kaur and Khurana, 2017; Marwaha, et al., 2005; Raigond et al., 2018. The storage behaviour of local potato cultivar PU1 was medium/poor (noted during this study).

Supplementary Table S2

List of the metabolites detected

S. No.	Metabolite	CID Code	Chemical shift	Assignment
1	3-hydroxyisobutyrate	87	1.07 (d)	β-CH_3
			2.47 (m)	α-CH
2	4-Aminobutyrate	119	1.89 (m)	β-CH_2
			2.29 (t)	α-CH_2
			3.00 (t)	γ-CH_2
3	Adenosine	60961	6.07 (d)	C_2H
4	Alanine	5950	3.80 (q)	α-CH
			1.46 (d)	β-CH_3
5	Allantoin	204	5.37 (s)	CH
6	Arginine	6322	1.68 (m)	γ-CH_2
			1.89 (m)	β-CH_2
			3.23 (t)	δ-CH_2
			3.76 (t)	α-CH
7	Ascorbate	54670067	4.51 (d)	C_4H
8	Asparagine	6267	2.84 (m)	β-CH_2
			2.94 (m)	β-CH_2
			3.99 (dd)	α-CH
9	Aspartate	5960	2.66 (dd)	β-CH_2
			2.79 (dd)	β-CH_2
			3.91 (dd)	α-CH
10	Chlorogenate	1794427	6.40 (d)	$C_{15}H$
			6.95 (d)	$C_{21}H$
			7.13 (dd)	$C_{22}H$
			7.19 (d)	$C_{18}H$
			7.66 (d)	$C_{16}H$
11	Choline	305	3.20 (s)	$N(CH_3)_3$
			3.52 (m)	NCH_2
			4.06 (m)	OCH_2
12	Citrate	311	2.67 (d)	CH_2
			2.76 (d)	CH_2
13	DSS	74873	0.00 (s)	$Si(CH_3)_3$

			0.62 (t)	γ-CH_2
			1.75 (m)	β-CH_2
			2.91 (t)	α-CH_2
14	Formate	283	8.44 (s)	CH
15	Fructose	2723872	3.54 (m)	C_1H
			3.70 (m)	C_1H
			3.88 (dd)	C_3H
			4.01 (m)	C_4H
			4.11 (d)	C_5H
16	Fumarate	5460307	6.50 (s)	CH
17	Galactose	6036	4.57 (d)	C_2H
			5.25 (d)	C_2H
18	Glucose	5793	3.23 (dd)	C_3H
			3.39 (m)	C_5H
			3.45 (m)	C_6H
			3.52 (m)	C_3H
			3.72 (m)	$C_4H/C_{11}H$
			3.82 (m)	$C_{11}H/C_6H$
			3.88 (dd)	$C_{11}H$
			4.63 (d)	C_2H
			5.22 (d)	C_2H
19	Glutamate	33032	2.03 (m)	β-CH_2
			2.10 (m)	β-CH_2
			2.34 (m)	γ-CH_2
			3.75 (dd)	α-CH
20	Glutamine	5961	2.14 (m)	β-CH_2
			2.459 (m)	γ-CH_2
			3.76 (t)	α-CH
21	Glycine	750	3.56 (s)	CH_2
22	Isoleucine	6306	0.92 (t)	δ-CH3
			0.99 (d)	β-CH_3
			1.24 (m)	γ-CH_2
			1.45 (m)	γ-CH_2
			1.97 (m)	β-CH

			3.66 (d)	α-CH
23	LDL		0.84 (t)	$CH_3(CH_2)_n$
			1.25 (m)	$(CH_2)_n$
24	Leucine	6106	0.94 (d)	δ-CH3
			0.95 (d)	δ-CH3
			1.68 (m)	β-CH2
			1.70 (m)	γ-CH
			3.71 (t)	α-CH
25	Lysine	5962	1.49 (m)	γ-CH_2
			1.72 (m)	δ-CH_2
			1.89 (m)	β-CH_2
			3.01 (t)	e-CH_2
			3.74 (t)	α-CH
26	Malate	525	2.37 (dd)	β-CH_2
			2.66 (dd)	β-CH_2
			4.29 (dd)	α-CH
27	Mannose	18950	4.89 (d)	C_2H
			5.17 (d)	C_2H
28	Methanol	887	3.34 (s)	CH_3
29	Methionine	6137	2.12 (s)	S-CH_3
			2.17 (m)	β-CH_2
			2.63 (t)	S-CH_2
			3.85 (t)	α-CH
30	Myo-inositol	892	3.27 (t)	C_5H
			3.54 (dd)	C_1H/C_3H
			3.62 (dd)	C_4H/C_6H
			4.05 (t)	C_2H
31	Phenylalanine	6140	7.42 (m)	C_3H/C_5H
			7.36 (m)	C_2H/C_6H
			7.32 (m)	C_4H
			3.98 (dd)	α-CH
			3.27 (m)	β-CH_2
32	Proline	145742	4.11 (t)	α-CH
			3.40 (m)	δ-CH_2
			3.33 (m)	δ-CH_2

			2.34 (m)	β-CH_2
			2.07 (m)	β-CH_2
			2.01 (m)	γ-CH_2
33	Pyroglutamate	7405	2.02 (m)	C_4H
			2.40 (m)	C_3H
			2.49 (m)	C_4H
			4.16 (m)	C_5H
34	Serine	5951	3.83 (m)	α-CH
			3.94 (m)	β-CH_2
			3.98 (m)	β-CH_2
35	sn-glycero-3-phosphocholine	657272	3.21 (s)	$C_{13}H/C_{14}H$
			4.31 (m)	C_3H
36	Sucrose	5988	3.46 (t)	$C_{10}H$
			3.55 (m)	$C_{12}H$
			3.75 (m)	$C_{11}H$
			3.81 (m)	$C_{17}H/C_{19}H$
			3.88 (m)	C_5H
			4.04 (t)	C_4H
			4.20 (d)	C_3H
			5.40 (d)	C_7H
37	Threonine	6288	1.32 (d)	γ-CH_3
			3.58 (d)	α-CH
			4.24 (m)	β-CH_2
38	Trigonelline	5570	4.42 (s)	C_9H
			8.07 (t)	C_4H
			8.82 (m)	C_5H/C_3H
			9.11 (s)	C_1H
39	Trypotophan	6305	3.29 (m)	CH_2
			4.05 (m)	CH
			7.19 (t)	C_5H/C_6H
			7.27 (t)	C_5H/C_6H
			7.31 (s)	C_2H
			7.53 (d)	C_7H

			7.72 (d)	C_4H
40	Tyrosine	6057	7.17 (d)	C_2H/C_6H
			6.87 (d)	C_3H/C_5H
			3.93 (dd)	α-CH
			3.18 (dd)	β-CH_2
			3.04 (dd)	β-CH_2
41	Uridine	6029	7.85 (d)	$C_{11}H$
			5.9 (d)	C_2H
			5.89 (d)	$C_{10}H$
			4.34 (dd)	C_3H
			4.22 (dd)	C_4H
			4.12 (m)	C_5H
			3.9 (dd)	$C_{14}H$
			3.8 (dd)	$C_{14}H$
42	Valine	6287	0.99 (d)	CH_3
			1.04 (d)	CH_3
			2.28 (m)	β-CH
			3.61 (d)	α-CH
43	U1		8.22 (m)	
			9.00 (d)	
			9.10 (d)	
			9.33 (s)	
44	U2		7.27 (s)	
			8.39 (s)	
45	U3		3.85	
			3.95	
			4.02	
			5.14 (d)	
46	U4		3.82	
			3.89	
			3.97	
			4.03	
			4.99	
47	U5		3.66	
			4.29 (dd)	

Supplementary Table S3: Person's correlation coefficient analysis was used to analyse the metabolite-metabolite correlation among identified metabolites in all five cultivars used in the study at two time points – fresh harvest and one month cold storage. The number of correlation observed for the metabolites in the different cultivars have been listed.

Cultivar – time point	Positive	Negative	Total
Atlantic FH	40	9	49
Atlantic CS	51	4	55
Frito Lay 1533 FH	46	5	51
Frito Lay 1533 CS	44	9	53
Kufri Pukhraj FH	31	5	36
Kufri Pukhraj CS	37	15	52
Kufri Jyoti FH	43	5	48
Kufri Jyoti CS	38	15	53
PU1 FH	42	3	45
PU1 CS	18	13	31

Supplementary Figures

Figure S: 1D ^{1}H-NMR spectrum of the methanolic extract of the Kufri Pukhraj potato cultivar (Cold storage) used in the study (as described in Materials and Methods). A combination of line-shape, multiplicity, scalar coupling, and chemical shift values obtained from this spectrum were used to identify 39 abundant metabolites. The assignment was validated with the BMRB and HMDB databases. The chemical shift and multiplicity details of the metabolic ensemble (marked in numbers) in the spectrum have been listed in Table S2.

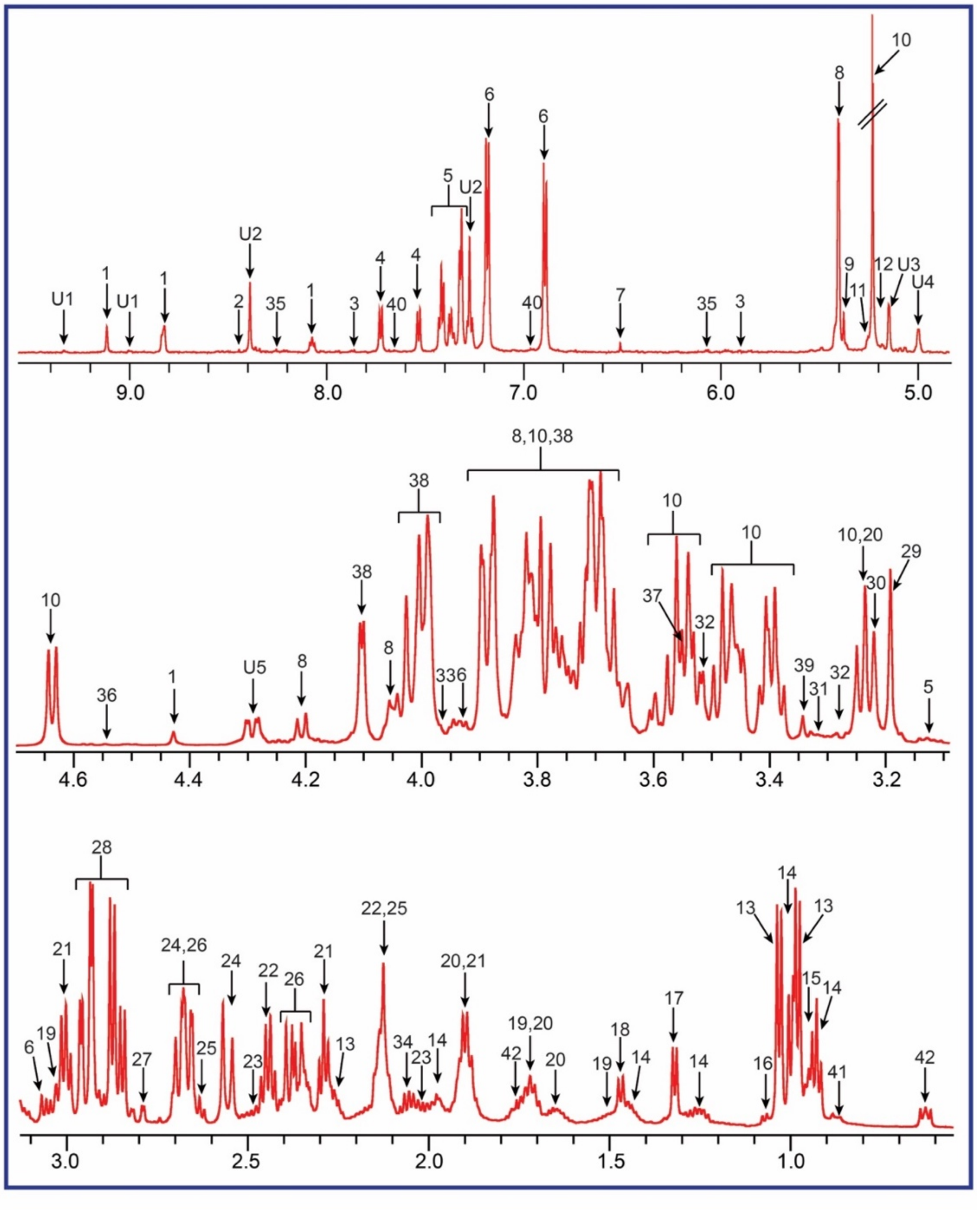

Figure S2: ^1H-^1H TOCSY correlation spectrum of the methanolic extract of fresh harvest of Kufri Pukhraj potato cultivar (cold storage) used in the study (as described in Materials and Methods). The cross-peaks in the TOCSY spectrum have been used to re-confirm the resonance assignments enlisted in Table S2.

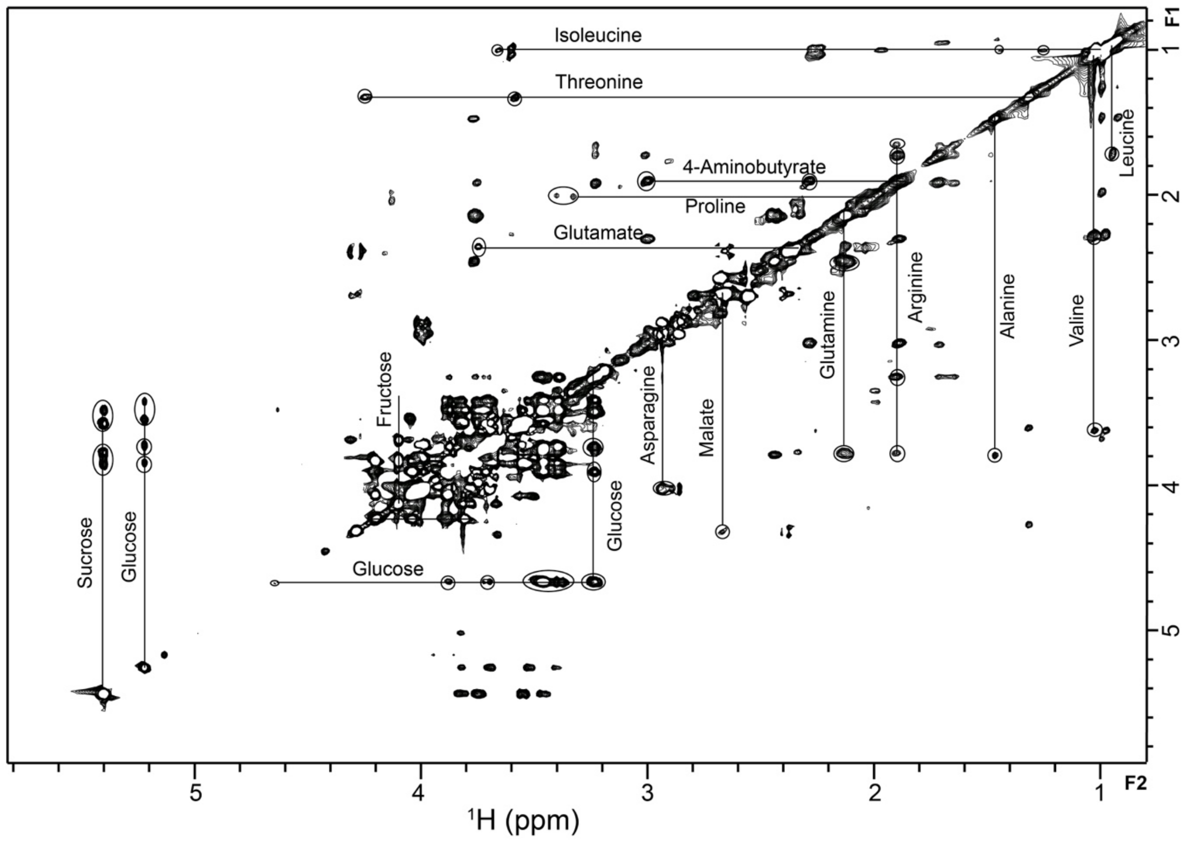

Figure S3: Scores plot as obtained by PCA utility of MetaboAnalyst software for the different potato cultivars – Atlantic (G1, G2), Frito Lay-1533 (G3, G4), Kufri Pukhraj (G5, G6), Kufri Jyoti (G7, G8), and PU1 (G9, G10) at fresh harvest and one-month cold storage at 4°C. Three replicates were used for each cultivar and at each condition (as described in Methods). Ellipses showing 95% confidence limits of a normal distribution for each group of the samples have been marked in respective colours for each cultivar. Colour legends have been mentioned in the figure.

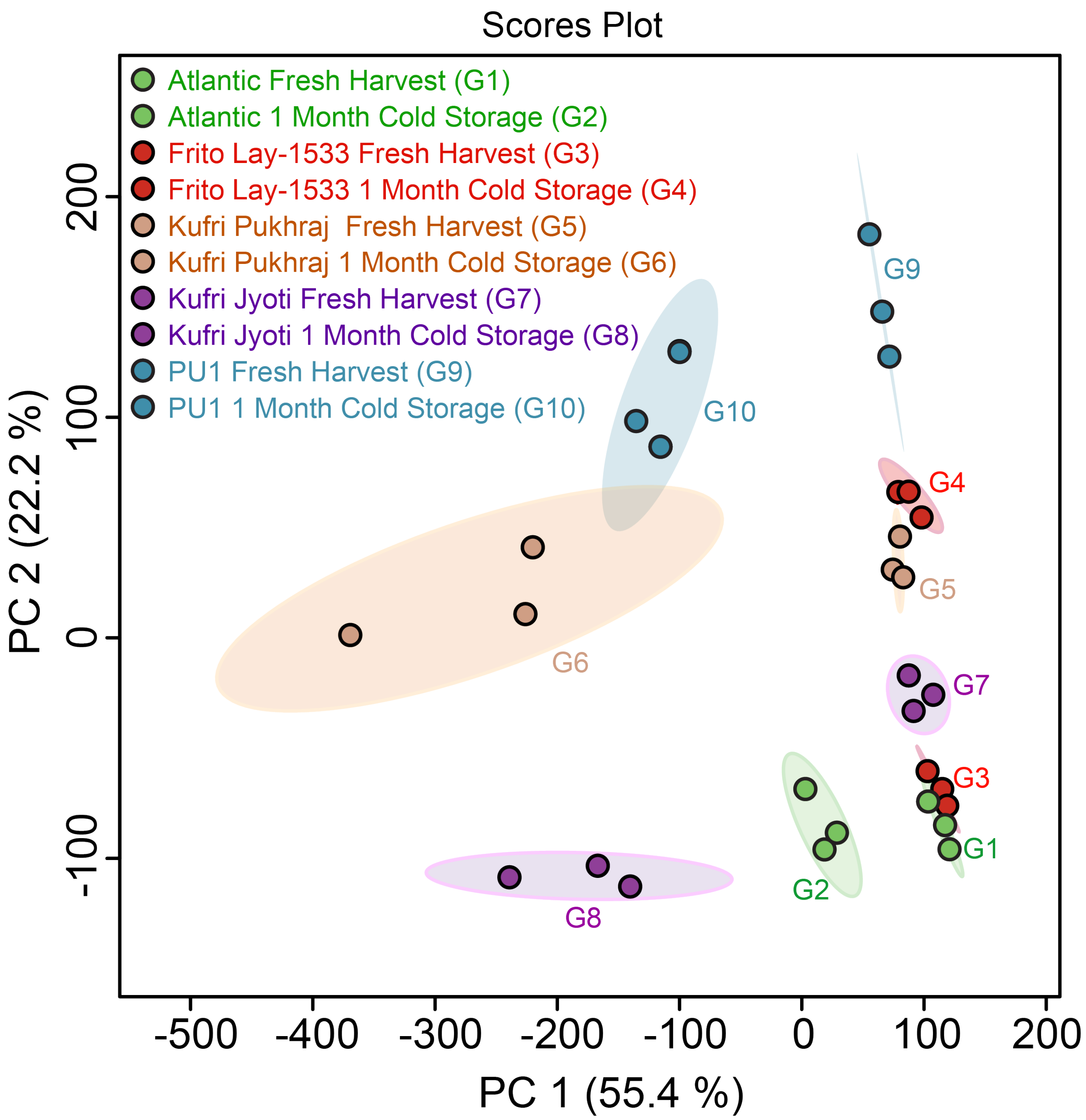

Figure S4: PLS-DA scores plots for pair-wise analysis of metabolites obtained from the different cultivars of potatoes at fresh harvest (Red) and cold storage at 4°C for 1 month (Green). A) Atlantic, B) Frito Lay-1533, C) Kufri Jyoti, D) Kufri Pukhraj, and E) PU1. Ellipses showing 95% confidence limits of a normal distribution for each group of the samples have been marked in respective colours (as mentioned above).

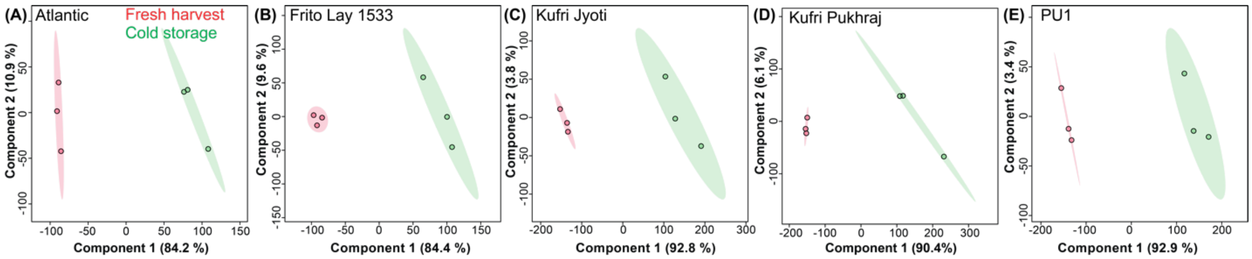

Figure S5: Correlation plots between fresh harvest (FH; upper-right half of the plot marked in white) and cold storage (CS; lower-left half of the plot marked in light blue) metabolites for potato tubers of Atlantic cultivar. Both the upper half and lower half diagonal correlation plots are independent of each other and must be read independently.

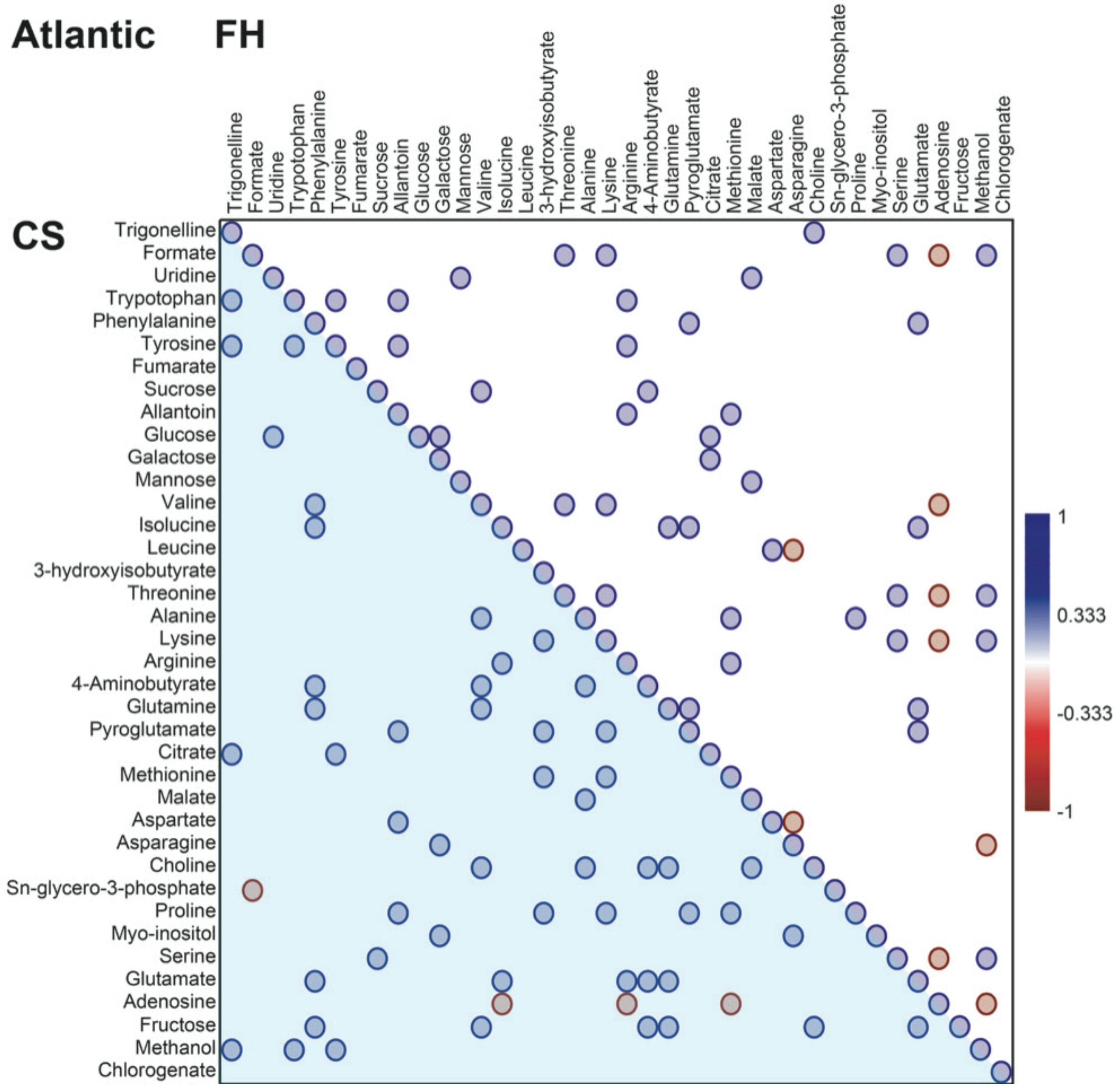

Figure S6: Correlation plots between fresh harvest (FH; upper-right half of the plot marked in white) and cold storage (CS; lower-left half of the plot marked in light blue) metabolites for potato tubers of FL-1533 cultivar. Both the upper half and lower half diagonal correlation plots are independent of each other and must be read independently.

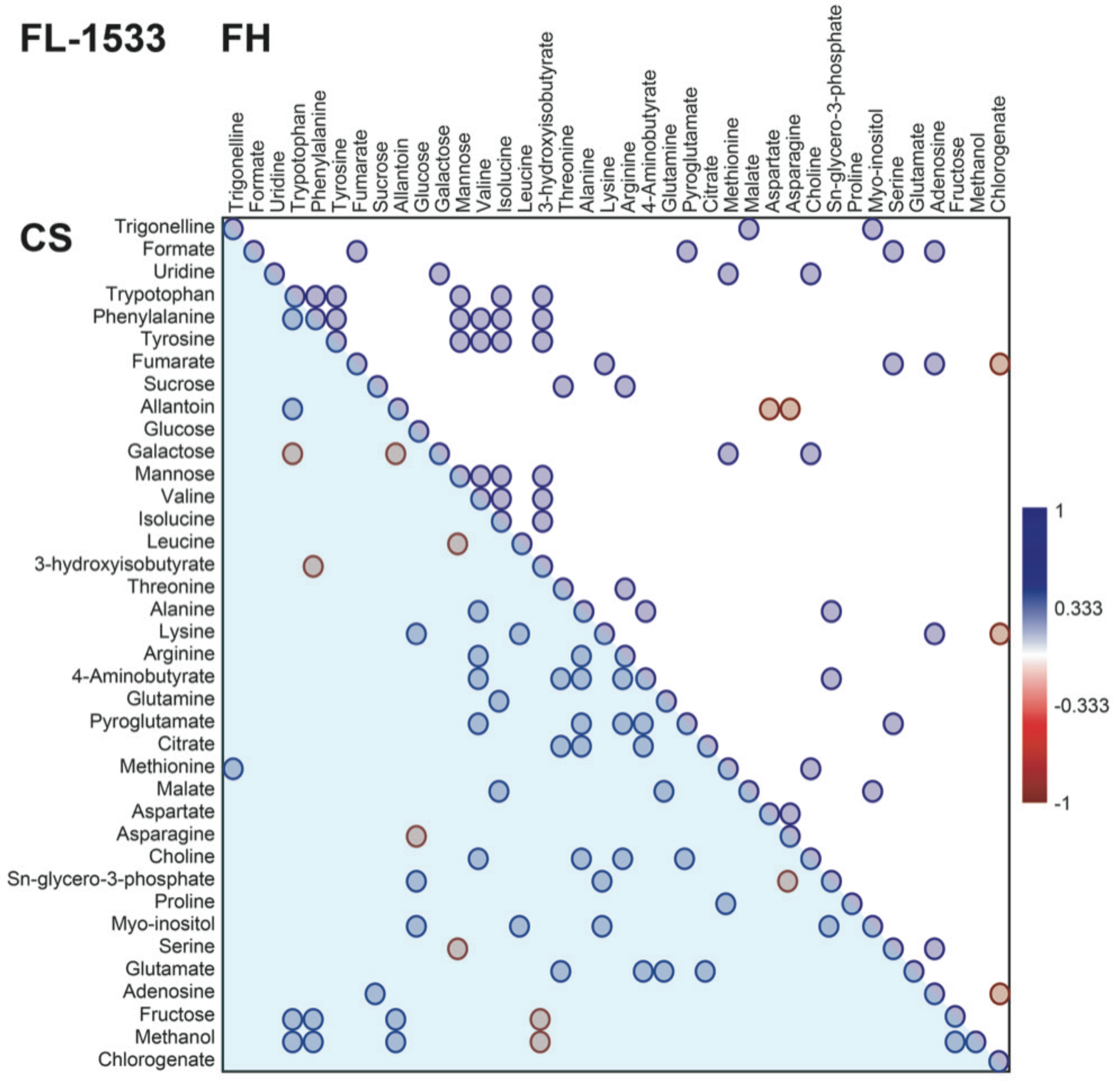

Figure S7: Correlation plots between fresh harvest (FH; upper-right half of the plot marked in white) and cold storage (CS; lower-left half of the plot marked in light blue) metabolites for potato tubers of Kufri Jyoti cultivar. Both the upper half and lower half diagonal correlation plots are independent of each other and must be read independently.

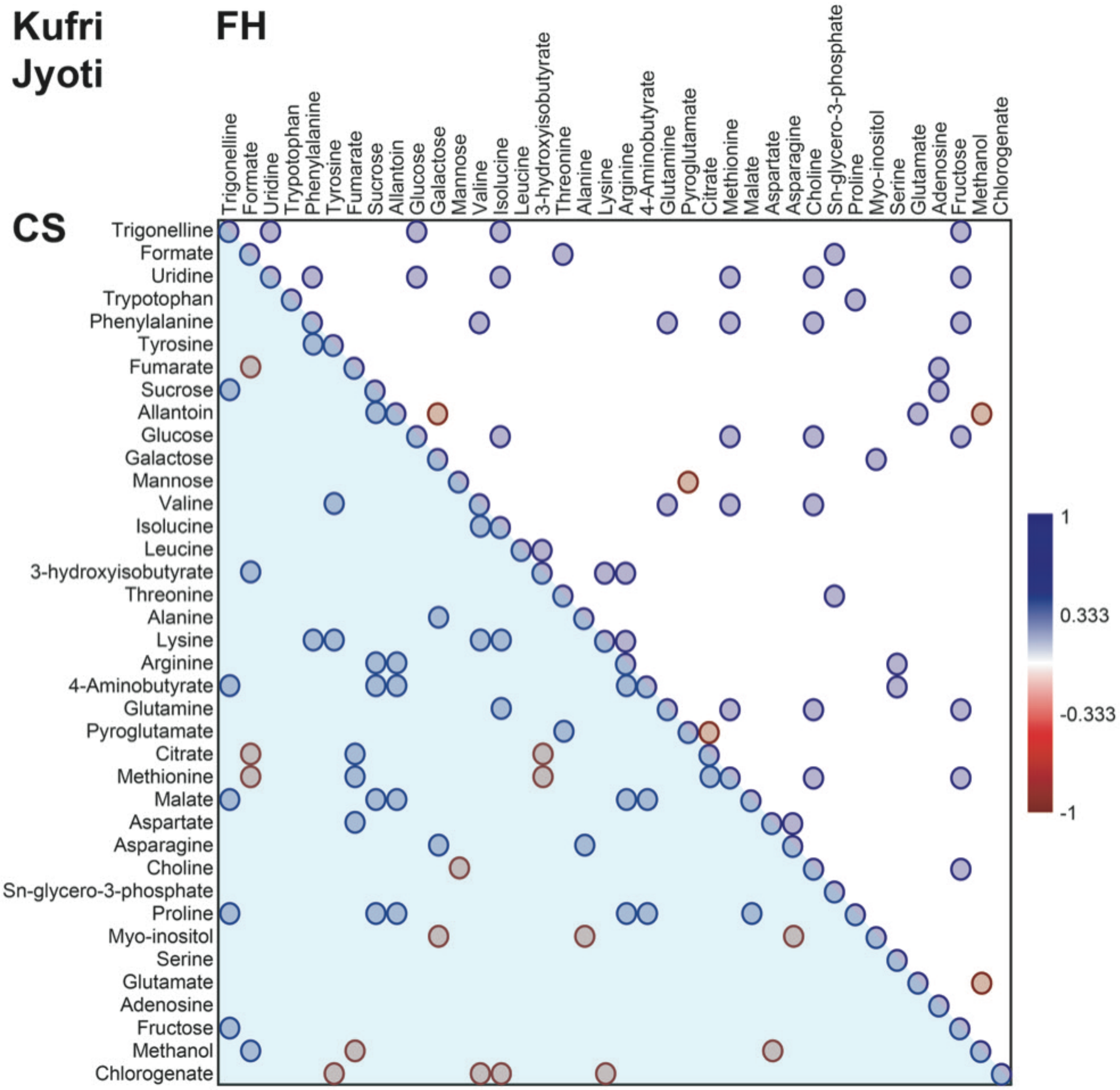

Figure S8: Correlation plots between fresh harvest (FH; upper-right half of the plot marked in white) and cold storage (CS; lower-left half of the plot marked in light blue) metabolites for potato tubers of Kufri Pukhraj cultivar. Both the upper half and lower half diagonal correlation plots are independent of each other and must be read independently.

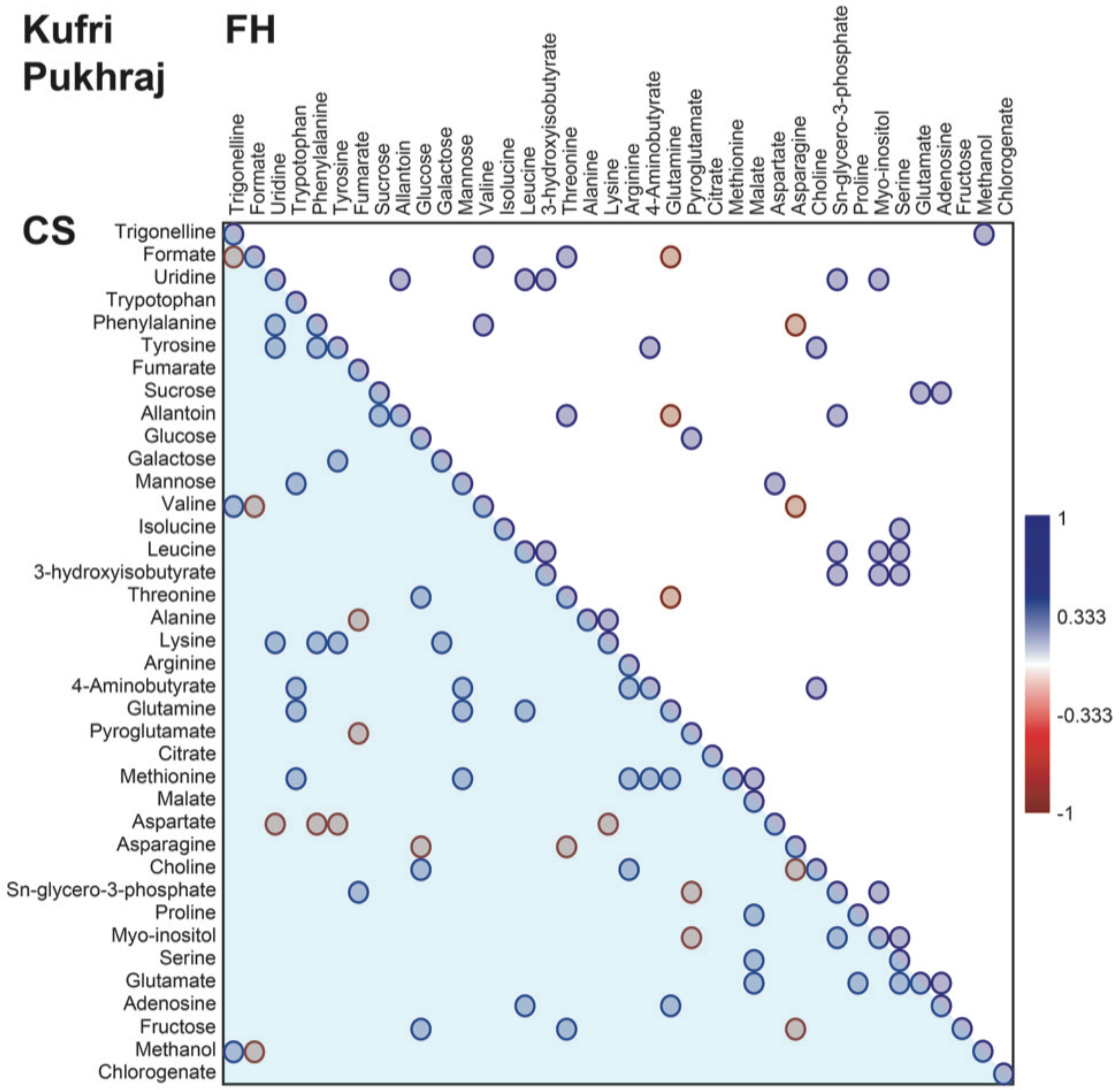

Figure S9: Correlation plots between fresh harvest (FH; upper-right half of the plot marked in white) and cold storage (CS; lower-left half of the plot marked in light blue) metabolites for potato tubers of PU1 cultivar. Both the upper half and lower half diagonal correlation plots are independent of each other and must be read independently.

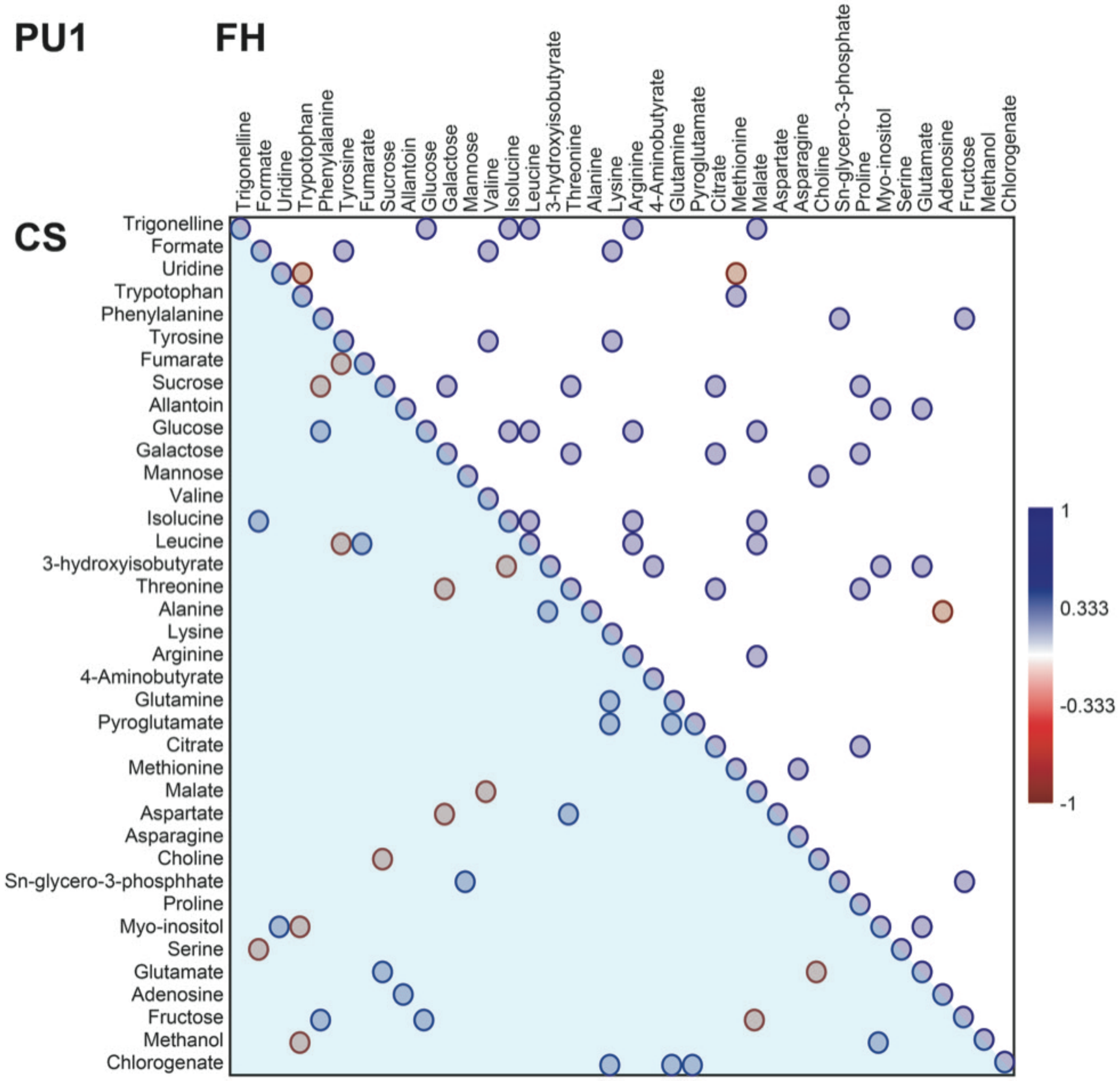

Supplement: Supplementary file 1 — Supplemental Information. [file 41598_2020_63329_MOESM1_ESM.pdf]
